# 2-Chloro-*N*′-[4-(dimethyl­amino)­benzyl­idene]-*N*-[4-(3-methyl-3-phenyl­cyclo­but­yl)-1,3-thia­zol-2-yl]acetohydrazide

**DOI:** 10.1107/S1600536810049962

**Published:** 2010-12-15

**Authors:** Ersin Inkaya, Muharrem Dinçer, Alaaddin Çukurovalı, Engin Yılmaz

**Affiliations:** a Department of Physics, Arts and Sciences Faculty, Ondokuz Mayıs University, 55139 Samsun, Turkey; b Department of Chemistry, Arts and Sciences Faculty, Fırat University, 23119 Elazığ, Turkey; c Department of Chemistry, Arts and Science Faculty, Bitlis Eren University, 13000 Bitlis, Turkey

## Abstract

The mol­ecular conformation of the title compound, C_25_H_27_ClN_4_OS, is stabilized by an intra­molecular benzyl­idine C—H⋯N_thia­zole_ hydrogen bond. The thiazole ring makes dihedral angles of 12.0 (3) and 20.4 (2)°, respectively, with the phenyl and benzene rings, while the phenyl and benzene rings make a dihedral angle of 22.6 (2)°. The crystal packing involves weak inter­molecular thia­zole C—H⋯O_carbon­yl_ and methyl C—H⋯π hydrogen-bonding associations.

## Related literature

For applications of related compounds, see: Brown *et al.* (1974 ▶); Dehmlow & Schmidt (1990 ▶); Foerster *et al.* (1979) ▶; Roger *et al.* (1977 ▶); Sawhney *et al.* (1978) ▶; Slip *et al.* (1974) ▶; Suzuki *et al.* (1979) ▶. For background to Schiff bases, see: Costamagna *et al.* (1992 ▶); Fita *et al.* (2005 ▶); Sridharan *et al.* (2004 ▶). For related structures, see: Dinçer *et al.* (2004 ▶); Demir *et al.* (2006 ▶); Özdemir *et al.* (2004 ▶); Soylu *et al.* (2005 ▶); Xu *et al.* (1994 ▶). For bond-length data, see: Allen (1984 ▶). 
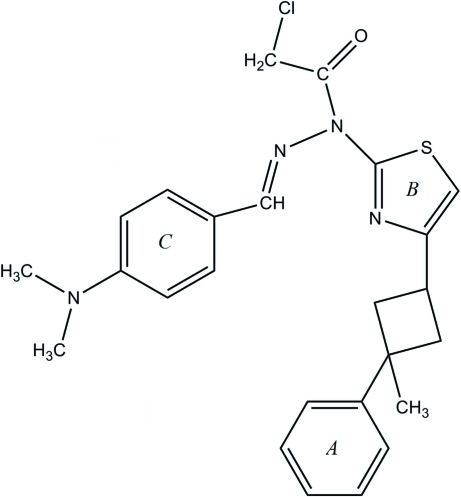


## Experimental

### 

#### Crystal data

C_25_H_27_ClN_4_OS
*M* *_r_* = 467.02
Monoclinic, 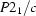

*a* = 9.0194 (5) Å
*b* = 26.7946 (11) Å
*c* = 13.1773 (7) Å
β = 132.054 (3)°
*V* = 2364.6 (2) Å^3^
*Z* = 4
Mo *K*α radiation
μ = 0.28 mm^−1^
*T* = 296 K
0.62 × 0.36 × 0.02 mm

#### Data collection

Stoe IPDS 2 CCD diffractometer
Absorption correction: integration (*X-RED32*; Stoe & Cie, 2002 ▶) *T*_min_ = 0.533, *T*_max_ = 0.896
22742 measured reflections
4446 independent reflections
2250 reflections with *I* > 2σ(*I*)
*R*_int_ = 0.143

#### Refinement

*R*[*F* ^2^ > 2σ(*F* ^2^)] = 0.070
*wR*(*F* ^2^) = 0.121
*S* = 1.01
4446 reflections
292 parameters
H-atom parameters constrained
Δρ_max_ = 0.19 e Å^−3^
Δρ_min_ = −0.17 e Å^−3^

### 

Data collection: *X-AREA* (Stoe & Cie, 2002 ▶); cell refinement: *X-AREA*; data reduction: *X-RED32* (Stoe & Cie, 2002 ▶); program(s) used to solve structure: *SHELXS97* (Sheldrick, 2008 ▶); program(s) used to refine structure: *SHELXL97* (Sheldrick, 2008 ▶); molecular graphics: *ORTEP-3 for Windows* (Farrugia, 1997 ▶); software used to prepare material for publication: *WinGX* (Farrugia, 1999 ▶) and *PLATON* (Spek, 2009 ▶).

## Supplementary Material

Crystal structure: contains datablocks global, I. DOI: 10.1107/S1600536810049962/zs2082sup1.cif

Structure factors: contains datablocks I. DOI: 10.1107/S1600536810049962/zs2082Isup2.hkl

Additional supplementary materials:  crystallographic information; 3D view; checkCIF report

## Figures and Tables

**Table 1:** Hydrogen-bond geometry (Å, °)

*D*—H⋯*A*	*D*—H	H⋯*A*	*D*⋯*A*	*D*—H⋯*A*
C17—H17⋯N1	0.93	2.21	2.838 (5)	124
C13—H13⋯O1^i^	0.93	2.50	3.374 (5)	157
C16—H16*A*⋯*Cg*1^ii^	0.97	2.57	3.493	159

Symmetry codes: (i) 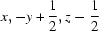; (ii) 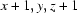.

**Fig. 1.**
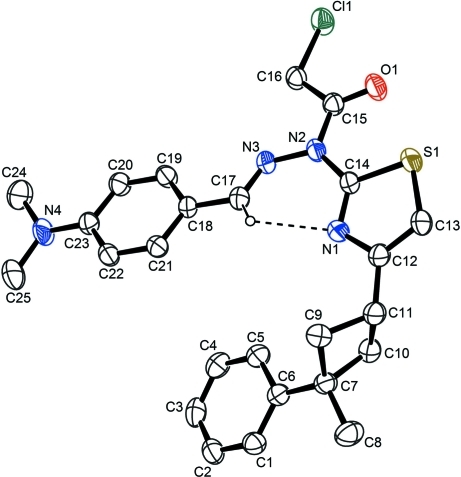
An ORTEP-3 (Farrugia, 1997) drawing of (I), showing the atomic numbering scheme. Displacement ellipsoids are drawn at the 30% probability level. The hydrogen bond is shown as a dashed line. For clarity, only H atoms involved in hydrogen bonding have been included.

**Fig. 2.**
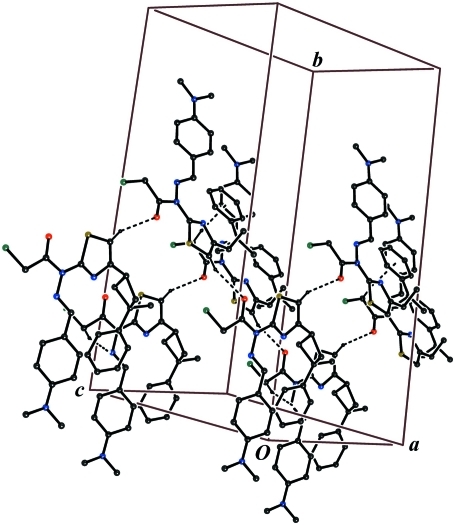
Part of the crystal structure of the title compound, showing the C—H···O and C—H···π interactions. For clarity, only H atoms involved in hydrogen bonding have been included. For symmetry codes, see Table 1.

## supplementary crystallographic information

### Comment

3-Substituted cyclobutane carboxylic acids exhibit anti-inflammatory and anti-depressant activity (Roger *et al.*,1977), as well as having liquid crystal properties (Dehmlow & Schmidt, 1990). Also, various thiazole derivatives have been shown to possess herbicidal (Foerster, *et al.*, 1979), anti-inflammatory (Sawhney *et al.*, 1978; Brown *et al.*, 1974), anti-microbial (Suzuki *et al.*,1979), and anti-parasitic properties (Slip *et al.*, 1974). Schiff bases are important in the development of coordination chemistry and Schiff base ligands are of interest mainly because of the existence of typical hydrogen bonds and tautomerism between the phenol–imine and keto–amine forms (Costamagna *et al.*, 1992; Sridharan *et al.*, 2004; Fita *et al.*, 2005). The synthesis and structure of the title compound, *N*-(4-dimethylaminobenzylidene)-*N*- [4-(3-methyl-3-phenyl-cyclobutyl)-thiazol-2-yl]-chloroacetic acid hydrazide, C_25_H_27_N_4_OClS (I) is reported here.

In the structure of (I) (Fig. 1) the phenyl and thiazole rings are *cis*-related with respect to the cyclobutane ring. The cyclobutane ring is puckered, with a dihedral angle of 22.99 (47)° between the two three-membered halves of the ring, which is more puckered than other similar examples from the literature, e.g. 11.55 (3)°, (Özdemir *et al.*, 2004) and 19.8 (3)° (Dinçer *et al.*, 2004). The dihedral angle between plane *A* (C1—C6), the thiazole plane *B* (N1/C14/S1/C13/C12) and the phenyl plane C (C18—C21) are 11.95 (25)° (*A/B*), 22.61 (23)° (*A/C*) and 20.36 (23)° (*B/C*), respectively. In the thiazole ring, the S1—C14 and S1—C13 bond lengths are 1.743 (4) Å and 1.707 (4) Å which are shorter than the accepted value for an S—C*sp^2^* single bond (1.76 Å; Allen, 1984) and is the result the conjugation of the electrons of atom S1 with atoms C14 and C13. The C—Cl and C═O bond distances are 1.779 (3) Å and 1.217 (4) Å, respectively, and these values are significantly shorter than those in the literature [1.807 (12) and 1.187 (16) Å, respectively (Demir *et al.*, 2006]. The C17═N3 bond length [1.276 (4) Å] compares with a literature value of 1.285 (7) Å (Xu *et al.*, 1994). In the thiazole ring the C12—N1 and C14═N1 bond lengths [1.389 (5) and 1.292 (4) Å, respectively] compare with literature values of 1.394 (4) and 1.339 (4)Å, respectively (Soylu *et al.*, 2005).

The conformation of the azide substituent ring systems of the title compound is stabilized by an intramolecular benzylidine C17—H···N1_thiazole_ hydrogen bond (Fig. 1, Table 1) and crystal packing involves weak intermolecular thiazole C13—H···O1_carbonyl_ and methyl C16—H···π (phenyl ring C1–C6) hydrogen-bonding associations (Fig. 2).

### Experimental

A solution of 1 mmol of chloroacetyl chloride in 10 ml of 1,4-dioxane was added to a mixture of 0.3905 g (1 mmol) of dimethyl-(4-{[4-(3-methyl-3-phenyl-cyclobutyl)-thiazol-2-yl]-hydrazonomethyl} -phenylamine and 1 mmol of triethylamine in 20 ml of 1,4-dioxane, at room temperature with continuous stirring. The course of the reaction was monitored by IR spectroscopy. The target product was precipitated with the slow addition of water, filtered, washed with copious cold ethanol and dried in air. The shiny crystals suitable for X-ray analysis was obtained by slow evaporation from an alcoholic solution. Yield: 83%, m.p. 420 K (EtOH). IR (KBr, ν cm^-1^): 2974–2813 (aliphatic), 1703 (C=O), 1612 (C=N thiazole), 728 (–CH_2_—Cl),634 (C—S). ^1^H NMR (CDCl_3_, TMS, δ, p.p.m.): 1.57 (s, 3H, –CH_3_, on cyclobutane), 2.50–2.65 (m, 4H, –CH_2_– on cyclobutane), 3.05 (s, 6H, –CH_3_ on aniline),3.77 (quint, j = 8.78 Hz, 1H, >CH– on cyclobutane),4.80 (s, 2H, –CH_2_—Cl),6.66 (d, j = 8.78 Hz, 2H, aromatic), 6.82 (s, 1H, =CH—S on thiazole), 7.14–7.21 (m, 3H, aromatics), 7.28 (t, j = 6.95 Hz, 2H, aromatic), 7.44 (d, j = 8.78 Hz, 2H, aromatic), 8.78 (s, 1H, –N═CH– azomethine). ^13^C NMR (CDCl_3_, TMS, δ, p.p.m.): 167.07, 156.56, 155.42, 152.71, 152.38, 129.88, 128.46, 125.50, 125.00, 120.90, 111.89, 111.30, 44.03, 41.21, 40.35, 38.95, 31.01, 30.10.

### Refinement

H atoms were positioned geometrically and treated using a riding model, fixing the bond lengths at 0.96, 0.97, 0.98 and 0.93 Å for CH_3_, CH_2_, CH and CH (aromatic), respectively. The displacement parameters of the H atoms were constrained with *U*_iso_(H) = 1.2*U*_eq_(aromatic, methylene or methine C) or 1.5*U*_eq_ (methyl C).

### Crystal data

**Table:**

C_25_H_27_ClN_4_OS	*F*(000) = 984
*M**_r_* = 467.02	*D*_x_ = 1.312 Mg m^−^^3^
Monoclinic, *P*2_1_/*c*	Melting point: 420 K
Hall symbol: -P 2ybc	Mo *K*α radiation, λ = 0.71073 Å
*a* = 9.0194 (5) Å	Cell parameters from 14801 reflections
*b* = 26.7946 (11) Å	θ = 1.5–26.2°
*c* = 13.1773 (7) Å	µ = 0.28 mm^−^^1^
β = 132.054 (3)°	*T* = 296 K
*V* = 2364.6 (2) Å^3^	Plate, brown
*Z* = 4	0.62 × 0.36 × 0.02 mm

### Data collection

**Table:**

Stoe IPDS 2 CCD diffractometer	4446 independent reflections
Radiation source: fine-focus sealed tube	2250 reflections with *I* > 2σ(*I*)
graphite	*R*_int_ = 0.143
Detector resolution: 6.67 pixels mm^-1^	θ_max_ = 25.6°, θ_min_ = 1.5°
rotation method scans	*h* = −10→10
Absorption correction: integration (*X-RED32*; Stoe & Cie, 2002)	*k* = −32→32
*T*_min_ = 0.533, *T*_max_ = 0.896	*l* = −16→16
22742 measured reflections	

### Refinement

**Table:**

Refinement on *F*^2^	Primary atom site location: structure-invariant direct methods
Least-squares matrix: full	Secondary atom site location: difference Fourier map
*R*[*F*^2^ > 2σ(*F*^2^)] = 0.070	Hydrogen site location: inferred from neighbouring sites
*wR*(*F*^2^) = 0.121	H-atom parameters constrained
*S* = 1.01	*w* = 1/[σ^2^(*F*_o_^2^) + (0.0287*P*)^2^] where *P* = (*F*_o_^2^ + 2*F*_c_^2^)/3
4446 reflections	(Δ/σ)_max_ < 0.001
292 parameters	Δρ_max_ = 0.19 e Å^−^^3^
0 restraints	Δρ_min_ = −0.17 e Å^−^^3^

### Special details

**Table:**

Geometry. All e.s.d.'s (except the e.s.d. in the dihedral angle between two l.s. planes) are estimated using the full covariance matrix. The cell e.s.d.'s are taken into account individually in the estimation of e.s.d.'s in distances, angles and torsion angles; correlations between e.s.d.'s in cell parameters are only used when they are defined by crystal symmetry. An approximate (isotropic) treatment of cell e.s.d.'s is used for estimating e.s.d.'s involving l.s. planes.
Refinement. Refinement of *F*^2^ against ALL reflections. The weighted *R*-factor *wR* and goodness of fit *S* are based on *F*^2^, conventional *R*-factors *R* are based on *F*, with *F* set to zero for negative *F*^2^. The threshold expression of *F*^2^ > σ(*F*^2^) is used only for calculating *R*-factors(gt) *etc*. and is not relevant to the choice of reflections for refinement. *R*-factors based on *F*^2^ are statistically about twice as large as those based on *F*, and *R*- factors based on ALL data will be even larger.

### Fractional atomic coordinates and isotropic or equivalent isotropic displacement parameters (Å^2^)

**Table:**

	*x*	*y*	*z*	*U*_iso_*/*U*_eq_	
Cl1	0.45265 (18)	0.13475 (4)	0.49349 (10)	0.0796 (4)	
N1	0.2748 (4)	0.14808 (12)	−0.0565 (3)	0.0585 (9)	
N2	0.3574 (4)	0.12803 (12)	0.1519 (3)	0.0563 (8)	
N3	0.3105 (4)	0.07697 (12)	0.1292 (3)	0.0552 (8)	
N4	0.1455 (6)	−0.15632 (14)	−0.0154 (4)	0.0862 (12)	
O1	0.4501 (5)	0.18872 (11)	0.3042 (3)	0.0788 (9)	
S1	0.43474 (18)	0.22208 (4)	0.11117 (10)	0.0743 (4)	
C1	−0.1810 (5)	0.05376 (17)	−0.5909 (3)	0.0609 (11)	
H1	−0.1777	0.0674	−0.6542	0.073*	
C2	−0.2743 (6)	0.00874 (17)	−0.6195 (4)	0.0700 (12)	
H2	−0.3308	−0.0079	−0.7004	0.084*	
C3	−0.2841 (6)	−0.01176 (17)	−0.5289 (5)	0.0764 (13)	
H3	−0.3498	−0.0419	−0.5488	0.092*	
C4	−0.1959 (6)	0.01272 (18)	−0.4082 (4)	0.0755 (13)	
H4	−0.2012	−0.0011	−0.3460	0.091*	
C5	−0.1001 (6)	0.05746 (17)	−0.3794 (4)	0.0661 (11)	
H5	−0.0396	0.0733	−0.2970	0.079*	
C6	−0.0916 (5)	0.07958 (15)	−0.4710 (3)	0.0529 (10)	
C7	0.0148 (5)	0.12898 (15)	−0.4359 (3)	0.0572 (10)	
C8	−0.0100 (6)	0.15109 (18)	−0.5534 (4)	0.0809 (14)	
H8A	0.0609	0.1822	−0.5249	0.121*	
H8B	−0.1491	0.1567	−0.6305	0.121*	
H8C	0.0427	0.1283	−0.5786	0.121*	
C9	0.2381 (5)	0.13060 (16)	−0.3001 (3)	0.0628 (11)	
H9A	0.2754	0.1036	−0.2377	0.075*	
H9B	0.3309	0.1330	−0.3138	0.075*	
C10	−0.0258 (5)	0.16916 (15)	−0.3724 (4)	0.0661 (11)	
H10A	−0.0808	0.1557	−0.3352	0.079*	
H10B	−0.1051	0.1970	−0.4330	0.079*	
C11	0.2018 (5)	0.18072 (15)	−0.2618 (3)	0.0617 (11)	
H11	0.2369	0.2089	−0.2896	0.074*	
C12	0.2851 (5)	0.18755 (15)	−0.1199 (3)	0.0596 (11)	
C13	0.3684 (6)	0.22920 (15)	−0.0435 (4)	0.0731 (12)	
H13	0.3875	0.2584	−0.0716	0.088*	
C14	0.3471 (5)	0.16094 (14)	0.0637 (3)	0.0543 (10)	
C15	0.4014 (5)	0.14549 (16)	0.2682 (3)	0.0563 (10)	
C16	0.3891 (6)	0.10708 (15)	0.3459 (3)	0.0599 (11)	
H16A	0.4799	0.0798	0.3731	0.072*	
H16B	0.2547	0.0937	0.2877	0.072*	
C17	0.3002 (5)	0.05152 (15)	0.0428 (4)	0.0593 (11)	
H17	0.3185	0.0671	−0.0112	0.071*	
C18	0.2602 (5)	−0.00161 (14)	0.0285 (3)	0.0507 (9)	
C19	0.2467 (6)	−0.02964 (16)	−0.0652 (3)	0.0650 (12)	
H19	0.2626	−0.0139	−0.1203	0.078*	
C20	0.2104 (6)	−0.08041 (16)	−0.0799 (4)	0.0677 (12)	
H20	0.2008	−0.0977	−0.1453	0.081*	
C21	0.2381 (5)	−0.02730 (16)	0.1094 (3)	0.0607 (11)	
H21	0.2465	−0.0097	0.1740	0.073*	
C22	0.2044 (6)	−0.07735 (16)	0.0970 (4)	0.0646 (11)	
H22	0.1921	−0.0929	0.1541	0.077*	
C23	0.1877 (5)	−0.10629 (16)	0.0009 (4)	0.0597 (11)	
C24	0.1179 (8)	−0.18412 (18)	−0.1206 (5)	0.1007 (17)	
H24A	0.0632	−0.2163	−0.1301	0.151*	
H24B	0.2443	−0.1881	−0.0961	0.151*	
H24C	0.0279	−0.1664	−0.2056	0.151*	
C25	0.1709 (7)	−0.18470 (19)	0.0877 (5)	0.1005 (17)	
H25A	0.3076	−0.1828	0.1720	0.151*	
H25B	0.1362	−0.2189	0.0593	0.151*	
H25C	0.0859	−0.1714	0.1008	0.151*	

### Atomic displacement parameters (Å^2^)

**Table:**

	*U*^11^	*U*^22^	*U*^33^	*U*^12^	*U*^13^	*U*^23^
Cl1	0.1198 (9)	0.0642 (8)	0.0901 (7)	0.0022 (7)	0.0849 (7)	−0.0016 (6)
N1	0.0693 (19)	0.050 (2)	0.0533 (17)	−0.0062 (16)	0.0399 (16)	−0.0047 (15)
N2	0.070 (2)	0.041 (2)	0.0609 (18)	−0.0027 (16)	0.0450 (17)	−0.0051 (15)
N3	0.0614 (19)	0.0405 (19)	0.0599 (18)	−0.0059 (15)	0.0391 (16)	−0.0039 (15)
N4	0.133 (3)	0.040 (2)	0.096 (3)	−0.015 (2)	0.081 (3)	−0.0071 (19)
O1	0.124 (2)	0.048 (2)	0.0852 (18)	−0.0129 (18)	0.0785 (18)	−0.0124 (15)
S1	0.1041 (8)	0.0462 (7)	0.0685 (6)	−0.0156 (6)	0.0561 (6)	−0.0103 (5)
C1	0.057 (2)	0.065 (3)	0.054 (2)	0.004 (2)	0.0342 (19)	0.000 (2)
C2	0.061 (3)	0.058 (3)	0.069 (3)	0.001 (2)	0.034 (2)	−0.011 (2)
C3	0.067 (3)	0.048 (3)	0.096 (3)	−0.001 (2)	0.047 (3)	0.004 (2)
C4	0.083 (3)	0.071 (3)	0.079 (3)	0.002 (3)	0.057 (3)	0.016 (2)
C5	0.076 (3)	0.064 (3)	0.062 (2)	−0.001 (2)	0.048 (2)	0.004 (2)
C6	0.052 (2)	0.059 (3)	0.053 (2)	0.007 (2)	0.0370 (19)	0.0055 (18)
C7	0.060 (2)	0.058 (3)	0.0501 (19)	−0.003 (2)	0.0356 (19)	0.0014 (18)
C8	0.087 (3)	0.083 (4)	0.065 (2)	−0.016 (3)	0.048 (2)	0.008 (2)
C9	0.063 (2)	0.067 (3)	0.056 (2)	−0.003 (2)	0.039 (2)	−0.0031 (19)
C10	0.071 (3)	0.053 (3)	0.065 (2)	−0.001 (2)	0.042 (2)	0.003 (2)
C11	0.069 (3)	0.052 (3)	0.057 (2)	−0.009 (2)	0.039 (2)	0.0017 (18)
C12	0.064 (2)	0.050 (3)	0.055 (2)	−0.008 (2)	0.035 (2)	0.0020 (18)
C13	0.098 (3)	0.043 (3)	0.069 (2)	−0.017 (2)	0.052 (2)	−0.0034 (19)
C14	0.058 (2)	0.042 (2)	0.054 (2)	−0.0026 (18)	0.0336 (19)	−0.0028 (17)
C15	0.064 (2)	0.052 (3)	0.060 (2)	0.002 (2)	0.044 (2)	−0.0001 (19)
C16	0.071 (3)	0.053 (3)	0.066 (2)	0.005 (2)	0.050 (2)	−0.0013 (18)
C17	0.070 (3)	0.045 (3)	0.062 (2)	0.000 (2)	0.045 (2)	0.0029 (19)
C18	0.055 (2)	0.041 (2)	0.0541 (19)	0.0010 (18)	0.0355 (18)	−0.0001 (17)
C19	0.089 (3)	0.051 (3)	0.058 (2)	−0.001 (2)	0.051 (2)	−0.0007 (19)
C20	0.099 (3)	0.048 (3)	0.065 (2)	−0.001 (2)	0.059 (2)	−0.0039 (19)
C21	0.072 (3)	0.053 (3)	0.063 (2)	−0.007 (2)	0.048 (2)	−0.0086 (19)
C22	0.078 (3)	0.054 (3)	0.069 (2)	−0.010 (2)	0.052 (2)	−0.001 (2)
C23	0.062 (2)	0.048 (3)	0.062 (2)	−0.002 (2)	0.038 (2)	−0.0016 (19)
C24	0.133 (4)	0.051 (3)	0.114 (4)	−0.012 (3)	0.081 (3)	−0.015 (3)
C25	0.127 (4)	0.065 (4)	0.111 (3)	−0.012 (3)	0.081 (3)	0.012 (3)

### Geometric parameters (Å, °)

**Table:**

Cl1—C16	1.779 (3)	C9—C11	1.545 (5)
N1—C14	1.292 (4)	C9—H9A	0.9700
N1—C12	1.389 (5)	C9—H9B	0.9700
N2—C15	1.382 (4)	C10—C11	1.558 (5)
N2—N3	1.404 (4)	C10—H10A	0.9700
N2—C14	1.412 (5)	C10—H10B	0.9700
N3—C17	1.276 (4)	C11—C12	1.487 (5)
N4—C23	1.371 (5)	C11—H11	0.9800
N4—C25	1.435 (5)	C12—C13	1.345 (5)
N4—C24	1.441 (6)	C13—H13	0.9300
O1—C15	1.217 (4)	C15—C16	1.508 (5)
S1—C13	1.707 (4)	C16—H16A	0.9700
S1—C14	1.743 (4)	C16—H16B	0.9700
C1—C2	1.371 (6)	C17—C18	1.450 (5)
C1—C6	1.385 (5)	C17—H17	0.9300
C1—H1	0.9300	C18—C19	1.379 (5)
C2—C3	1.370 (6)	C18—C21	1.392 (5)
C2—H2	0.9300	C19—C20	1.382 (5)
C3—C4	1.378 (6)	C19—H19	0.9300
C3—H3	0.9300	C20—C23	1.396 (5)
C4—C5	1.374 (6)	C20—H20	0.9300
C4—H4	0.9300	C21—C22	1.360 (5)
C5—C6	1.392 (5)	C21—H21	0.9300
C5—H5	0.9300	C22—C23	1.405 (5)
C6—C7	1.514 (5)	C22—H22	0.9300
C7—C8	1.528 (5)	C24—H24A	0.9600
C7—C10	1.550 (5)	C24—H24B	0.9600
C7—C9	1.560 (5)	C24—H24C	0.9600
C8—H8A	0.9600	C25—H25A	0.9600
C8—H8B	0.9600	C25—H25B	0.9600
C8—H8C	0.9600	C25—H25C	0.9600
			
C14—N1—C12	110.6 (3)	C12—C11—H11	110.9
C15—N2—N3	112.8 (3)	C9—C11—H11	110.9
C15—N2—C14	120.9 (3)	C10—C11—H11	110.9
N3—N2—C14	126.1 (3)	C13—C12—N1	114.2 (3)
C17—N3—N2	122.9 (3)	C13—C12—C11	127.0 (4)
C23—N4—C25	121.3 (4)	N1—C12—C11	118.8 (3)
C23—N4—C24	120.5 (4)	C12—C13—S1	112.0 (3)
C25—N4—C24	116.9 (4)	C12—C13—H13	124.0
C13—S1—C14	87.95 (19)	S1—C13—H13	124.0
C2—C1—C6	122.3 (4)	N1—C14—N2	122.9 (3)
C2—C1—H1	118.9	N1—C14—S1	115.2 (3)
C6—C1—H1	118.9	N2—C14—S1	121.8 (3)
C3—C2—C1	119.9 (4)	O1—C15—N2	121.0 (4)
C3—C2—H2	120.0	O1—C15—C16	123.8 (3)
C1—C2—H2	120.0	N2—C15—C16	115.2 (3)
C2—C3—C4	119.4 (4)	C15—C16—Cl1	110.0 (3)
C2—C3—H3	120.3	C15—C16—H16A	109.7
C4—C3—H3	120.3	Cl1—C16—H16A	109.7
C5—C4—C3	120.3 (4)	C15—C16—H16B	109.7
C5—C4—H4	119.9	Cl1—C16—H16B	109.7
C3—C4—H4	119.9	H16A—C16—H16B	108.2
C4—C5—C6	121.5 (4)	N3—C17—C18	120.2 (4)
C4—C5—H5	119.3	N3—C17—H17	119.9
C6—C5—H5	119.3	C18—C17—H17	119.9
C1—C6—C5	116.6 (4)	C19—C18—C21	116.4 (4)
C1—C6—C7	123.4 (3)	C19—C18—C17	121.1 (4)
C5—C6—C7	120.0 (3)	C21—C18—C17	122.5 (4)
C6—C7—C8	113.4 (3)	C18—C19—C20	122.1 (4)
C6—C7—C10	116.2 (3)	C18—C19—H19	118.9
C8—C7—C10	110.5 (4)	C20—C19—H19	118.9
C6—C7—C9	116.2 (3)	C19—C20—C23	121.6 (4)
C8—C7—C9	110.6 (3)	C19—C20—H20	119.2
C10—C7—C9	87.2 (3)	C23—C20—H20	119.2
C7—C8—H8A	109.5	C22—C21—C18	122.0 (4)
C7—C8—H8B	109.5	C22—C21—H21	119.0
H8A—C8—H8B	109.5	C18—C21—H21	119.0
C7—C8—H8C	109.5	C21—C22—C23	122.2 (4)
H8A—C8—H8C	109.5	C21—C22—H22	118.9
H8B—C8—H8C	109.5	C23—C22—H22	118.9
C11—C9—C7	90.3 (3)	N4—C23—C20	122.3 (4)
C11—C9—H9A	113.6	N4—C23—C22	122.1 (4)
C7—C9—H9A	113.6	C20—C23—C22	115.6 (4)
C11—C9—H9B	113.6	N4—C24—H24A	109.5
C7—C9—H9B	113.6	N4—C24—H24B	109.5
H9A—C9—H9B	110.9	H24A—C24—H24B	109.5
C7—C10—C11	90.2 (3)	N4—C24—H24C	109.5
C7—C10—H10A	113.6	H24A—C24—H24C	109.5
C11—C10—H10A	113.6	H24B—C24—H24C	109.5
C7—C10—H10B	113.6	N4—C25—H25A	109.5
C11—C10—H10B	113.6	N4—C25—H25B	109.5
H10A—C10—H10B	110.9	H25A—C25—H25B	109.5
C12—C11—C9	118.6 (3)	N4—C25—H25C	109.5
C12—C11—C10	116.1 (3)	H25A—C25—H25C	109.5
C9—C11—C10	87.5 (3)	H25B—C25—H25C	109.5
			
C15—N2—N3—C17	−167.2 (3)	C11—C12—C13—S1	177.2 (3)
C14—N2—N3—C17	18.0 (5)	C14—S1—C13—C12	0.9 (3)
C6—C1—C2—C3	1.1 (6)	C12—N1—C14—N2	−179.4 (3)
C1—C2—C3—C4	−1.4 (7)	C12—N1—C14—S1	−0.1 (4)
C2—C3—C4—C5	0.5 (7)	C15—N2—C14—N1	−168.1 (4)
C3—C4—C5—C6	0.9 (7)	N3—N2—C14—N1	6.4 (6)
C2—C1—C6—C5	0.2 (6)	C15—N2—C14—S1	12.6 (5)
C2—C1—C6—C7	179.1 (4)	N3—N2—C14—S1	−172.9 (3)
C4—C5—C6—C1	−1.2 (6)	C13—S1—C14—N1	−0.5 (3)
C4—C5—C6—C7	179.8 (4)	C13—S1—C14—N2	178.8 (3)
C1—C6—C7—C8	7.8 (5)	N3—N2—C15—O1	178.1 (4)
C5—C6—C7—C8	−173.3 (4)	C14—N2—C15—O1	−6.8 (6)
C1—C6—C7—C10	137.5 (4)	N3—N2—C15—C16	−0.4 (4)
C5—C6—C7—C10	−43.6 (5)	C14—N2—C15—C16	174.7 (3)
C1—C6—C7—C9	−122.0 (4)	O1—C15—C16—Cl1	0.7 (5)
C5—C6—C7—C9	56.8 (5)	N2—C15—C16—Cl1	179.2 (3)
C6—C7—C9—C11	−134.4 (3)	N2—N3—C17—C18	177.1 (3)
C8—C7—C9—C11	94.4 (4)	N3—C17—C18—C19	179.4 (3)
C10—C7—C9—C11	−16.5 (3)	N3—C17—C18—C21	−2.1 (6)
C6—C7—C10—C11	134.3 (3)	C21—C18—C19—C20	0.9 (6)
C8—C7—C10—C11	−94.7 (3)	C17—C18—C19—C20	179.5 (4)
C9—C7—C10—C11	16.3 (3)	C18—C19—C20—C23	−0.8 (6)
C7—C9—C11—C12	134.9 (3)	C19—C18—C21—C22	−0.1 (6)
C7—C9—C11—C10	16.4 (3)	C17—C18—C21—C22	−178.7 (4)
C7—C10—C11—C12	−137.2 (3)	C18—C21—C22—C23	−0.7 (6)
C7—C10—C11—C9	−16.5 (3)	C25—N4—C23—C20	164.4 (4)
C14—N1—C12—C13	0.8 (5)	C24—N4—C23—C20	−1.5 (7)
C14—N1—C12—C11	−177.7 (3)	C25—N4—C23—C22	−17.7 (7)
C9—C11—C12—C13	140.8 (4)	C24—N4—C23—C22	176.3 (4)
C10—C11—C12—C13	−117.0 (5)	C19—C20—C23—N4	177.9 (4)
C9—C11—C12—N1	−40.9 (5)	C19—C20—C23—C22	0.0 (6)
C10—C11—C12—N1	61.3 (5)	C21—C22—C23—N4	−177.2 (4)
N1—C12—C13—S1	−1.2 (5)	C21—C22—C23—C20	0.8 (6)

### Hydrogen-bond geometry (Å, °)

**Table:**

*D*—H···*A*	*D*—H	H···*A*	*D*···*A*	*D*—H···*A*
C17—H17···N1	0.93	2.21	2.838 (5)	124.
C13—H13···O1^i^	0.93	2.50	3.374 (5)	157.
C16—H16A···Cg1^ii^	0.97	2.57	3.493	159

Symmetry codes: (i) *x*, −*y*+1/2, *z*−1/2; (ii) *x*+1, *y*, *z*+1.
